# Supplementary material for: Are carbon emissions trading and green financial instruments synergistic? -Comprehensive quantitative research based on content analysis
Source: PLoS One. 2024 Mar 7;19(3):e0298601. doi: 10.1371/journal.pone.0298601 (PMC10919601; doi:10.1371/journal.pone.0298601)
Supplement: S1 Data — (ZIP) [file pone.0298601.s001.zip › Data/地级市Z_F工作报告.zip!/║╙─╧/─╧╤⌠╩╨/└┤╫╘╣1⁄2╓┌║┼í╛┬φ┐╦╩2╛▌═°í┐_2019.docx]

各位代表：

　　现在，我代表省人民政府，向大会报告工作，请予审议，并请各位政协委员和其他列席人士提出意见。

　　一、2018年工作回顾

　　过去的一年，面对严峻复杂的国际国内环境和艰巨繁重的改革发展任务，在省委领导下，全省上下以习近平新时代中国特色社会主义思想为指导，全面落实党的十九大精神和习近平总书记视察指导河南时的重要讲话精神，坚持稳中求进工作总基调，以新发展理念为引领，以高质量发展为根本方向，以供给侧结构性改革为主线，深入开展三大攻坚战，统筹推进“四个着力”，持续打好“四张牌”，全省经济社会发展保持了“稳、进、好”态势，实现了“三个同步”“三个高于”目标，高质量发展取得新成绩，中原更出彩迈出新步伐。

　　（一）牢固抓住关键环节，经济保持平稳增长。统筹推进稳增长、促改革、调结构、惠民生、防风险，全省经济平稳运行、稳中向好。全年生产总值48055.86亿元，比上年增长7.6%。

　　积极扩大有效需求。加大重点领域投资力度，“三山一滩”贫困地区高速公路和郑万、郑阜、太焦、郑济、商合杭高铁建设顺利推进，宿鸭湖水库清淤扩容、卫河共渠治理、青电入豫等重点工程开工建设；百城建设提质工程全面推进，实施项目8893个、投资6150亿元；全社会固定资产投资增长8%。鼓励社会力量进入教育、文化、医疗、康养等领域，着力满足新的消费需求，社会消费品零售总额增长10.3%。积极改善出口服务，引导企业开拓市场，出口总额增长12.8%。

　　培育壮大实体经济。推动资源要素向实体经济集聚、政策措施向实体经济倾斜、工作力量向实体经济加强，主动帮助实体企业解决实际困难。着力减轻企业负担，全年减免企业税收480亿元左右，降低用能等各种成本222.5亿元。打破民营企业发展壁垒，消除对非公经济歧视，出台系列扶持政策，持续改善营商环境，激发实体经济活力。全年新设立企业34.3万户、增长14.9%，实有市场主体590.6万户、稳居中部首位。全省规模以上工业增加值增长7.2%，主营业务收入超过百亿元的企业达到46家。

　　强化生产要素保障。帮助企业搭建融资对接平台，发挥三级担保体系作用，扩大周转还贷资金规模，探索供应链融资，促进企业从银行、保险、证券、基金等多渠道融资，融资总额超过9000亿元。举办首届招才引智创新发展大会，延揽了一批急需紧缺人才，开展职业技能培训337万人次。采取用好增量、盘活存量、加大流量措施，重点工程建设和企业生产用地需求得到有效保障。

　　（二）推进结构优化升级，发展质量效益提高。瞄准高质量发展主攻方向，深化供给侧结构性改革，加快建设现代化经济体系，着力提升供给质量效率。全年财政总收入5875.8亿元，一般公共预算收入3763.9亿元、增长10.5%，其中税收占70.6%，比上年提高2.2个百分点。

　　推进供给结构改革。持续压减低端和无效供给，在前两年化解过剩产能基础上，又化解煤炭产能825万吨、钢铁产能157万吨，淘汰落后煤电机组107.7万千瓦，压减电解铝低效产能35万吨。加大国有企业去杠杆力度，新落地市场化债转股183亿元，省管工业企业资产负债率下降1.3个百分点。加快壮大新产业、发展新业态、培育新模式，新能源汽车、服务机器人产量分别增长70.4%、37.8%，网络零售额和快递业务收入均增长30%以上。

　　促进产业转型升级。着眼高端化、智能化、绿色化、融合化发展目标，持续实施转型攻坚，以装备制造、新型材料、能源化工、绿色食品、电子信息、建筑装配、现代物流、文化旅游、健康养老、高效种养、烟草、酒业等12个重点产业为突破口，大力推进制造业绿色、智能、技术三大改造，创建国家级绿色工厂28家，培育建设智能工厂（车间）150家，工业技改投资增长21.5%，工业五大主导产业、高技术产业和战略性新兴产业增加值分别增长7.7%、12.3%和12.2%。社会物流总额预计增长9%，旅游总收入增长20.3%，服务业增加值占生产总值比重达到45.2%。

　　促进经济良性循环。开展质量提升和对标达标专项行动，大力推进创新制造、精品制造、品牌制造，精深加工产品和终端产品占比继续提高，省级产品质量监督抽查合格率达96.8%。漯河市以开展标准化建设引领高质量发展的做法受到国务院通报表扬。推进内贸流通体制改革，启动物流标准化示范建设，全省规模以上工业产销率达到98.7%。引导金融机构加大对重点产业支持力度，大力发展普惠金融、科技金融、绿色金融，人民币各项贷款余额增长14.6%。

　　（三）大力推进三大攻坚，努力补齐发展短板。坚持把打好三大攻坚战作为破解瓶颈制约、全面建成小康社会的硬任务，系统谋划，统筹安排，强力推动，实现良好开局。

　　防范化解重大风险。建立完善信贷风险补偿机制，帮助企业化解股权质押平仓风险，发挥债权人委员会作用，妥善处置企业债券刚性兑付和担保连带风险。去杠杆、治乱象、抓规范、强监管，有效防范金融风险。在全国率先逐县逐项开展政府隐性债务摸底审计调查，科学制定实施意见，采取“八个一批”方式积极稳妥有序化解，地方债务风险总体可控。切实防控非洲猪瘟，有效阻断重大动物疫病扩散蔓延。

　　扎实开展精准脱贫。坚持精准方略，科学制定脱贫攻坚战三年行动计划，组织开展“四场硬仗”“六大行动”和“四项工程”，把握攻坚重点，扎实有效推进。聚焦深度贫困县村、特殊贫困群体、重点贫困县区，强化对口帮扶，加大政策支持，改善发展条件，提高脱贫质量。贫困地区农民人均可支配收入增速高于全省农村平均水平1.6个百分点左右。易地扶贫搬迁提前完成“十三五”安置点住房建设任务，改造农村危房18.58万户，贫困县、贫困村电网脱贫改造任务提前全面完成，全年又有121.7万农村贫困人口脱贫，33个贫困县有望摘帽。黄河滩区居民迁建第二批试点3.8万人即将喜迁新居。

　　大力推进污染防治。召开全省生态环境保护大会，积极实施污染防治攻坚战三年行动计划，把大气污染防治作为重中之重抓住不放，实行科学治污、依法治污、全民治污，强化“六控”推进源头预防、调整“四个结构”加强综合治理、精准发力实施重点管控、完善制度建立长效机制，完成“电代煤”“气代煤”112.4万户，PM2.5、PM10平均浓度分别下降1.6%、2.8%。全面推行河长制湖长制，开展河道采砂综合治理。实施水资源、水生态、水环境、水灾害“四水同治”，启动十项重大水利工程，受到国务院通报表扬。开展土壤污染治理与修复试点，土壤环境质量总体保持稳定。围绕“五年增绿山川平原，十年建成森林河南”目标，按照“三增四转五统六化”总体要求，推进生态建设，完成造林260万亩，森林抚育改造450万亩，超额完成年度任务。

　　（四）深化改革开放创新，不断增强发展活力。着力破除体制机制障碍，充分激发市场活力，增强微观主体动力。

　　坚定不移推进改革。深化国有企业改革攻坚，按照市场化、法治化原则，采用清算注销、兼并重组、改制转让、盘活脱困、依法破产等方式，处置“僵尸企业”1124家；深入推进产权结构、组织结构、治理结构改革，盘活了闲置低效资产，增添了企业发展活力，优化了国有资本布局，完善了现代企业制度，得到中央领导充分肯定。深化政府“放管服”改革，“一网通办”前提下“最多跑一次”改革全面提速，省市县审批服务事项网上可办率均达到90%以上。深化投融资体制改革，推广投资项目告知承诺、容缺办理、多评合一等模式，投资项目审批事项由113项精简到59项，中原股权交易中心挂牌企业突破4500家。省级机构改革基本完成，机构设置和职能配置得到优化，市县机构改革有序启动。财税、金融、土地、环保、医疗、教育、文化、综合执法等领域改革稳步推进。

　　不断提升开放水平。中国（河南）自由贸易试验区政务、监管、金融、法律、多式联运五大服务体系加快建设，口岸功能不断提升，开放平台进一步完善。持续推动空中、陆上、网上、海上丝绸之路“四路”协同，开放通道进一步畅通。大幅压缩外商投资备案手续办理时间和进出口贸易整体通关时间，完善国际贸易“单一窗口”功能，落实出口退税政策，开放环境进一步优化。开放型经济发展呈现较好势头，郑州机场旅客吞吐量2733万人次、增长12.5%，货邮吞吐量51.5万吨，跨境电商交易额增长25%左右，全省进出口总值5512.7亿元，实际吸收外资179亿美元，引进省外资金9647亿元。

　　强力推进科技创新。加快郑洛新国家自主创新示范区管理体制和人事薪酬制度改革，发挥国家创新平台辐射带动效应。聚焦“四个一批”汇聚创新资源，推进“四个融合”开展协同创新，实施“十百千”转型升级创新专项，提升科技创新能力。16项成果荣获国家科技奖励。全国首个生物育种产业创新中心落户河南，新增高新技术企业突破1000家。万人发明专利拥有量增长17.2%，技术合同成交额149.7亿元、实现倍增。开工建设青年人才公寓5.14万套。航空港双创示范基地受到国务院督查激励。

　　（五）加快推进城乡建设，促进城乡共同发展。统筹实施乡村振兴战略和城市转型发展，城乡发展更趋协调。

　　实施乡村振兴战略。按照产业兴旺、生态宜居、乡风文明、治理有效、生活富裕的总要求，制定乡村振兴战略实施意见、规划和专项行动计划。围绕农业强，调结构、提质量、增动力，加快推进“四优四化”。粮食生产再获丰收，优质专用小麦、优质花生、优质草畜、优质林果规模继续扩大。基本完成农村承包地确权登记颁证，农村集体产权制度改革进展顺利，新型农业经营主体发展到28万家。开展“大棚房”专项清理整治。驻马店市借力农产品加工业会展经济助推大发展的做法受到国务院通报表扬。围绕农村美，加强生态环境保护，抓好人居环境整治，搞好村庄环境美化。新改造农村户用卫生厕所200万户，新建标准化村卫生室1371个、综合文化中心17672个，新增农村敬老院床位2.76万张。围绕农民富，提高农民收入水平，改善农民生活条件，增强农民保障能力。实现20户以上自然村4G网络全覆盖，新改建农村公路1万多公里，新安、兰考等6县获评“四好农村路全国示范县”。全省农村居民人均可支配收入增长8.7%，高于城镇0.9个百分点。

　　推进百城建设提质。以文明理念为引领，以人民群众为中心，以品质提升为重点，持续推进百城建设提质和文明城市创建。承载能力不断增强，新建改造城市道路1352公里、燃气管网5800公里、热力管网397公里、电力线路3453公里。人居环境不断优化，棚改安置房开工66.33万套、基本建成38.42万套，基本消除城市县城黑臭水体78处，新改建城市公厕3268座，新建游园351处。公共服务不断改善，加大政务、教育、卫生、文化、体育、养老等公共服务供给，办事难、就学难、看病难、养老难等一大批民生难题逐步得到解决。文明形象不断提升，又有22个市县入选全国文明城市提名城市，文明城市数量居全国第四位，文明城市创建覆盖面达到81.3%。越来越多的城市正在成为居者心怡、来者心悦的宜居之城。

　　加快中原城市群发展。完成郑州大都市区空间规划编制，明确“一核四轴三带多点”空间格局。支持郑州建设国家中心城市，新能源及网联汽车基地等重大产业工程、贾鲁河综合治理等重大生态项目全面实施，郑州市生产总值有望超过1万亿元。推动开封、新乡、焦作、许昌与郑州深度融合，郑开双创走廊、开港、许港等重点产业带建设全面推进，以轨道交通和城际快速路网为主体的大都市区通勤圈加快构建。支持洛阳建设副中心城市，洛炼扩能改造、格力智能装备等一批现代产业项目加快建设，伊洛瀍涧四河同治成效显著，城市水系、路网、绿廊加快形成。因城施策，分类指导，房地产市场运行总体平稳。常住人口城镇化率达到51.71%，提高1.55个百分点。

　　（六）切实保障改善民生，社会大局保持稳定。坚持以民生凝聚民心，着力解决关系群众切身利益问题，全年财政民生支出7126亿元，12项重点民生实事落地见效。

　　着力扩大社会就业。抓好高校毕业生、退役军人就业创业，做好“僵尸企业”处置和过剩产能化解中的职工安置、困难行业企业稳岗就业工作，深入推进返乡创业国家级试点，承办“中国创翼”创业创新大赛。城镇新增就业139万人，新增农村劳动力转移就业56万人，返乡下乡创业新增23万人、带动就业223万人，城镇登记失业率3.02%。居民人均可支配收入增长8.9%。

　　完善社会保障体系。建立企业养老保险基金省级调剂制度，完善机关事业单位养老保险制度，实现跨省异地就医直接结算定点医疗机构县级全覆盖。企业和机关事业单位退休人员基本养老金年人均分别提高1627元和1584元，城乡居民基础养老金最低标准年人均提高216元，城乡低保、居民医保财政补助标准持续提高。

　　积极发展社会事业。坚持教育优先发展，新建改扩建幼儿园1442所，新增城镇义务教育学位78.94万个，新建改扩建农村寄宿制学校903所，完成7146所贫困地区薄弱中小学校办学条件改善任务，五年“全面改薄”目标顺利实现。校外培训机构专项治理初见成效。高中阶段教育加快普及，中职学校布局调整任务圆满完成。郑州大学、河南大学“双一流”建设启动实施，全省新增博士学位授权点38个。分级诊疗制度逐步完善，基层诊疗能力持续提升，心血管、儿童国家区域医疗中心影响力增强。艺术创作成果丰硕，基层公共文化服务体系加快建设，严厉打击盗掘破坏殷墟文物等行为，文化遗产保护力度持续加大。成功举办省第十三届运动会，全民健身蓬勃开展。

　　推动社会治理创新。深入开展扫黑除恶专项斗争，依法打击各类违法犯罪和暴力恐怖活动，深化“一村（格）一警”工作，加强困难群体法律援助，群众安全感不断上升。严格落实安全生产责任制，深化重点行业领域安全专项治理，安全生产形势总体平稳。推进食品安全省建设，食品药品安全监管继续加强。农民工工资“治欠保支”力度持续加大。依法做好信访工作，信访总量明显下降，社会大局保持稳定。

　　加强国防教育和国防后备力量及人民防空建设，支持军队改革发展，推进军民融合深度发展，驻豫解放军、武警部队、预备役人员、民兵为地方发展作出新贡献，双拥工作持续深化。民族宗教工作创新推进。妇女儿童、残疾人、红十字、慈善等事业健康发展。外事、侨务、港澳、对台、统计、参事、史志、文史、地质、气象、测绘、地震、社科研究、援疆等工作取得新成绩。

　　我们自觉接受人大监督、政协民主监督和社会监督，认真办理人大代表建议和政协提案。加强法治政府建设，推进依法行政，强化审计监督，持续纠正“四风”，政府自身建设和治理能力不断提升。

　　过去的一年，看似平常如往、波澜不惊，实则形势复杂、挑战严峻，在实践中我们更加深切地体会到：第一，必须坚持党的集中统一领导。维护党中央权威和集中统一领导是我国制度优势的根本。中国经济是一艘巨轮，体量越大，风浪越大，掌舵领航至关重要。去年，美国挑起贸易大战，国际形势异常复杂，经济下行压力加大，一些问题水落石出。在面临诸多两难或多难选择的情况下，以习近平同志为核心的党中央高瞻远瞩、运筹帷幄，统揽“两个大局”，保持战略定力，采取正确策略，实施相机调控，推动中国经济这艘巨轮行稳致远。我们贯彻落实党中央、国务院决策部署，根据经济运行中出现的新矛盾和新问题，抓重点、补短板、强弱项、稳增长，突出抓好“四个稳定”。强化要素保障、落实税收抵扣、降低企业成本、增加信贷支持，稳定工业增长，工业产值和实现利润均保持较快增速；改善出口服务、加快出口退税，稳定对外贸易，全年出口逆势增长；加快工程项目建设、推动百城建设提质、积极应对楼市变化、着力激活民间投资，稳定有效投资，社会固定资产投资保持平稳增长；加强就业服务、开展技能培训、鼓励返乡创业、落实稳岗补贴，稳定社会就业，城镇新增就业超额完成年度目标。随着一系列稳增长措施落地见效，全省经济避免了大起大落，实现了稳中有进、持续向好。实践证明，党中央集中统一领导是我们应对重大挑战、抵御重大风险、克服重大阻力、化解重大矛盾、夺取伟大胜利的根本保证。我们必须牢固树立“四个意识”、坚定“两个维护”，自觉坚持党中央集中统一领导，确保党中央各项决策部署落到实处、见到实效。第二，必须树立防患未然底线思维。习近平总书记指出，预判风险所在是防范风险的前提，把握风险走向是谋求战略主动的关键。去年初，我们就组织力量对发现和潜在的风险隐患进行全面排查，重点盯住经济发展、政治安全、社会稳定、自然灾害、公共事件、安全生产六大领域，科学制定应对预案，严格落实部门责任，有备无患，防患未然。围绕防范经济领域风险，全面排查清理问题企业、问题项目、问题楼盘、问题工程，妥善处置企业股权质押平仓和债券兑付违约风险，推进市场化债转股，有效盘活了不良资产，阻断了风险传导，降低了企业风险；依法治理金融乱象，规范金融交易行为，严厉打击非法集资，整顿政府融资平台，确保了金融安全稳健运行；在全国率先开展地方隐性债务审计调查，弄清债务隐在哪、险在哪和化解难在哪、招在哪，摸清了“底子”，开出了“方子”，探索了“路子”，坚持既化解存量又严控增量，既抓紧“刹车”又防止“翻车”，既堵住“后门”又开好“前门”，既规范政策又分类施策，确保了债务风险总体可控，得到了中央领导的肯定。随着防范经济风险组合措施有效实施，守住了不发生系统性区域性风险的底线，稳定了企业经营，维护了河南形象，增强了市场信誉，提振了发展信心。与此同时，我们坚持严格依法，按照“四宜四不”原则，组织协调、指导漯河成功处置非法聚集群体事件，避免了冲突发生。提前开展抢险演练，加强气象监测预报，适时启动应急响应，全力防范台风灾害，有效减轻了“温比亚”带来的损失。实践证明，凡事预则立，不预则废，管住风险就有了发展基础，引爆风险就会失去发展条件。我们必须增强忧患意识，树立底线思维，既要有防范风险的先手，也要有应对和化解风险挑战的高招，打好化险为夷、转危为机的主动战，为经济社会发展奠定坚实的基础。第三，必须砥砺坚韧不拔改革意志。习近平总书记指出，改革开放是坚持和发展中国特色社会主义的必由之路，是决定当代中国命运的关键一招。过去的一年，我们始终坚持解放思想，开拓进取，扎实推进行政服务、商事制度、医药卫生、科技体制等各项改革。特别是针对全省国有企业发展活力不够、转型动力不足、创新能力不强的突出问题，我们按照党中央、国务院的部署，2016年打响了国有企业改革攻坚战，制定了一年“前哨战”、一年“总攻战”、一年“扫荡战”的时间表和路线图。在完成涉及百万职工家庭的企业办社会职能剥离基础上，去年我们深化企业产权结构、组织结构、治理结构改革，加快千家“僵尸企业”处置，或许一家“僵尸企业”就是一个“雷区”，不踩无险，踩了可能险象环生；或许一家“僵尸企业”就是一个“蜂窝”，不捅无事，捅了可能问题缠身，但是我们不畏难、不避险，坚韧不拔、矢志不渝，因企施策、一企一策，多方联动、一抓到底，啃下了“硬骨头”，打赢了“总攻战”，使一批曾经陷入窘境的企业拨云见日、柳暗花明，全省国有及国有控股企业实现利润增长70%以上。同时，我们深入推进自贸区先行先试、探索路子，以开放倒逼改革，以创新支撑发展，着力增强发展新动能，提升区域经济新优势。实践证明，改革开放是河南发展的不竭动力，也是破解问题的唯一选择，面临的矛盾越多、遇到的困难越大，越要砥砺改革意志、锤炼改革品格，自觉做到改革不停顿、开放不止步，以改革的精神来克服前进中的困难，用改革的办法来解决发展中的矛盾。第四，必须发扬迎难而上担当精神。习近平总书记指出，我们现在所处的，是一个船到中流浪更急、人到半山路更陡的时候，是一个愈进愈难、愈进愈险而又不进则退、非进不可的时候。过去的一年，在外部经济环境严峻和内部结构矛盾凸显的双重压力下，既要稳增长，防止经济过快下滑，影响发展预期，动摇增长信心，又要调结构，解决增长方式粗放、环境污染严重、质量效益不高的问题，我们正确把握稳增长与调结构的辩证统一，着力破解当前增长稳不住和长远发展保不了的难题，以“功成不必在我”的精神境界和“功成必定有我”的历史担当，迎着困难上、奔着问题去，坚定不移推进供给侧结构性改革、推动高质量发展。坚决“去”，在能源原材料价格上涨情况下，毫不含糊大幅压减煤炭、钢铁、电解铝、水泥等行业落后产能；积极“调”，调高技术水平、调强制造能力、调优产业结构、调长产业链条；加快“转”，下大力气摆脱过度依赖投资拉动的发展模式、过度依赖工业支撑的发展格局、过度依赖物质消耗的发展路径；大力“改”，扎实推进绿色改造、智能改造、技术改造，推动经济结构加快转型、发展方式加快转变、增长动能加快转换，实现了经济效益、生态效益、社会效益同步改善的综合效果。实践证明，事不避难、知难不难，矛盾和问题不会因为回避而自然消失，只能因为勇于正视才会逐步解决。我们必须以“越是艰险越向前”的英雄气概和“狭路相逢勇者胜”的斗争精神，一往无前，披荆斩棘，攻克前进道路上的一切艰难险阻，开拓河南发展更加光明的前景。第五，必须保持勤勉重干务实作风。习近平总书记指出，伟大梦想不是等得来、喊得来的，而是拼出来、干出来的。中原大地曾经写下了绚丽多彩的历史篇章，孕育了勤勉重干的务实作风，涌现了焦裕禄、史来贺、杨贵和吴金印等一大批先进模范人物，创造了弥足珍贵的精神财富。过去的一年，一批重点民生实事的办好，一批事关全局大事的办成，一批制约发展难事的办妥，特别是防范化解重大风险、精准脱贫、污染防治三大攻坚战取得新进展、实现新突破、开创新局面，都是全省广大干部群众勤勉肯干、务实重干的结果。面对脱贫攻坚艰巨任务，全省上下以锲而不舍的耐心和毅力、舍我其谁的气魄和担当、敢打硬仗的决心和意志，尽锐出战、苦干实干，在精准帮扶上出实招、在精准推进上鼓实劲、在精准落地上求实效，探索了卢氏金融扶贫、焦作健康扶贫、驻马店助残扶贫、南阳洛阳三门峡易地搬迁扶贫、兰考产业扶贫等叫响全国的经验做法，超额完成了年度脱贫任务。尤其是身在扶贫一线的基层干部，以对群众的亲劲、抓工作的韧劲、干事业的拼劲，谱写了战天斗地拔穷根、不破楼兰终不还的动人篇章。实践证明，一分耕耘、一分收获，正是大家抓铁有痕、踏石留印，撸起袖子加油干，扑下身子抓落实，才干出了新业绩、创出了新成就。我们必须发扬“钉钉子”精神，一锤接着一锤敲，一件接着一件干，靠实干成就事业，用实干铸就辉煌。第六，必须凝聚亿万人民创造伟力。习近平总书记指出，人民是历史的创造者，是决定党和国家前途命运的根本力量。过去的一年，我们之所以能取得这样的成绩，关键在于人民的创造伟力。以改革激发人民创造伟力，通过改革行政体制，“放”出活力、“管”出公平、“服”出便利，优化了发展环境，推动了创新创业；改革企业体制，使企业成为独立的经济实体和竞争主体，干部能上能下、职工能进能出、工资能增能降，激发了企业活力，增强了内生动力；改革科技体制，让科研人员有了更多机会、更好环境、更大舞台从事创新创造，众多改革打破了体制机制束缚，激发了亿万人民的创造热情、创造精神、创造活力。以发展鼓舞人民创造伟力，坚持在发展中共享、在共享中发展，努力使每个人既是发展的受益者又是发展的推动者，在百城提质和文明创建、环境治理和生态建设的实践中，人民群众既有“人改造环境、环境改造人”的获得感，又有“城市是我家，建设靠大家”的责任感，以主人翁的态度、建设者的担当参与到物质文明、精神文明、生态文明建设中来，汇聚了共建共享的强大力量。以事业凝聚人民创造伟力，为投资者营造环境，为创业者拓展空间，为劳动者搭建平台，始终让中原更加出彩成为亿万人民的奋斗追求和前进动力，在出彩的历史长卷上共同绘彩、添彩、喝彩，一股股建设河南的力量加快汇聚，一道道增辉中原的色彩正在绽放。正是有了亿万人民的创造伟力，在复杂形势、严峻挑战面前，我们才能攻坚克难、奋勇向前。实践证明，依靠人民群众，就会所向披靡、无往不胜；离开人民群众，就会寸步难行、一事无成。我们必须践行党的根本宗旨，贯彻党的群众路线，尊重人民主体地位，发挥人民首创精神，保障人民合法权益，汇聚亿万人民磅礴力量，绘就中原更加出彩崭新画卷。

　　回顾过去的一年，深感成绩来之不易。这是在省委领导下，全省人民以习近平新时代中国特色社会主义思想为指引，拼搏进取、团结奋斗的结果，离不开历届领导班子打下的坚实基础，离不开中央驻豫单位的共同参与，离不开中央和省各大媒体的大力支持。在此，我代表省人民政府，向全省各族人民，向驻豫人民解放军、武警部队官兵，向各民主党派、工商联、各人民团体及各界人士致以崇高的敬意！向关心支持河南发展的港澳台同胞、海外侨胞、国际友人表示诚挚的感谢！

　　在看到成绩的同时，我们也清醒认识到，经济社会发展中还面临不少困难和问题：一是转型发展任务艰巨。增长方式粗放、结构矛盾突出、创新能力不强的状况尚未根本改变，新产业、新业态、新模式依然较少，发展新动能仍然不足。二是企业经营困难较多。经济下行压力加大，市场需求增长放缓，能源原材料、人工、用地成本较高，企业盈利空间受限，部分企业经营困难加重。三是风险隐患不容忽视。市场预期不稳，一些企业面临再融资受阻、资金链断裂风险，个别市县偿还债务压力增大，经济社会矛盾交织，风险隐患不可小觑。四是民生还有不少短板。城乡居民持续增收制约因素较多，贫困群众生活改善任务较重，生态环境形势依然严峻，教育、医疗、养老等公共服务还不能很好满足人民群众需要。同时，政府工作存在不足，有些改革和政策措施落实不到位，一些干部观念落后、作风浮漂、能力不够，存在推拖庸懒、不作为慢作为现象。一些领域不正之风和腐败问题依然时有发生。对此，我们一定高度重视、切实解决，努力把各项工作做得更好。

　　二、2019年工作安排

　　2019年是新中国成立70周年，是全面建成小康社会、实现第一个百年奋斗目标的关键之年，做好今年的工作意义重大。

　　今年政府工作要认真落实省委十届八次全会要求：以习近平新时代中国特色社会主义思想为指导，全面贯彻党的十九大和十九届二中、三中全会及中央经济工作会议精神，持续落实习近平总书记视察指导河南时的重要讲话精神，统筹推进“五位一体”总体布局，协调推进“四个全面”战略布局，坚持稳中求进工作总基调，坚持新发展理念，坚持推动高质量发展，坚持以供给侧结构性改革为主线，坚持深化市场化改革、扩大高水平开放，贯彻巩固、增强、提升、畅通的方针，深入开展三大攻坚战，持续推进“四个着力”、打好“四张牌”，统筹推进稳增长、促改革、调结构、惠民生、防风险、保稳定工作，保持经济运行在合理区间，进一步稳就业、稳金融、稳外贸、稳外资、稳投资、稳预期，提振市场信心，增强人民群众获得感、幸福感、安全感，保持经济持续健康发展和社会大局稳定，为全面建成小康社会收官打下决定性基础，在中原更加出彩的征程中迈出更大步伐，以优异成绩庆祝中华人民共和国成立70周年。

　　今年经济社会发展主要预期目标是：继续保持“三个同步”“三个高于”，生产总值增长7%—7.5%，全员劳动生产率增长7%，一般公共预算收入增长7%，居民消费价格指数涨幅控制在3%左右，经济结构进一步优化，质量效益明显提升。创新发展取得新成果，研究与试验发展经费投入强度达到1.5%，高技术产业、战略性新兴产业增加值占规模以上工业比重分别达到10%以上和20%左右。协调发展取得新成效，常住人口城镇化率提高1.5个百分点以上，人均预期寿命持续提高，九年义务教育巩固率达到95%，医疗卫生、文化、体育等社会事业全面进步。绿色发展取得新提升，单位生产总值能耗下降3%以上，主要污染物排放量完成国家下达目标。开放发展取得新突破，进出口保持平稳增长，营商环境在全国评价排序前移2位以上。共享发展取得新进步，居民人均可支配收入增长8%，65万农村贫困人口实现脱贫，城镇调查失业率和城镇登记失业率分别控制在5.5%以内、4%以内。以上目标，更加凸显了新发展理念和高质量发展要求，有利于把工作重点放在推进高质量发展上来。

　　工作中，着力把握“四个关系”：一是从“形”与“势”的统一中审视全局，坚定必胜信心。当前，外部不确定因素增多，全省经济运行下行压力明显加大，但从长期大势看当前经济形势，河南发展的动力源是足的，投资、消费、出口、创新需求空间广阔；基本面是好的，反映经济运行的各项指标匹配性都很好；增长点是有的，企业适应市场的韧性普遍增强。特别是随着供给侧结构性改革的推进，支撑高质量发展的条件不断提升。我们必须辩证地审视大局，看趋势，观大势，防止被短期变化牵着鼻子走，既要看到“形”有波动，更要看到“势”仍向好，进一步坚定信心、稳定预期。二是从“危”与“机”的转换中抢抓机遇，赢得发展主动。当今世界正面临百年未有之大变局，既带来不利影响，也带来难得机遇，既要看到面临的“危”，也要看到面对的“机”。“危”和“机”历来相伴而生，失去了机即是危，克服了危就是机。历史经验证明，抓住了机遇必定会赢得发展，失去了机遇就可能耽误发展。要紧扣重要战略机遇期新内涵，危中寻机，化危为机，牢牢把握发展主动权，在应对困难和挑战中实现经济社会发展新的跃升。三是从“供”与“需”的重构中深化改革，培育壮大优势。当前经济发展面临的问题，供给和需求两侧都有，但矛盾的主要方面在供给侧。必须坚持以供给侧结构性改革为主线不动摇，把握供与需的平衡性、增强供与需的适应性、提高供与需的灵活性、强化供与需的畅通性，不断改善供给市场、提高供给质量，实现供需循环畅通高效，培育壮大发展优势。四是从“稳”与“进”的把握中迎难而上，保持战略定力。稳中求进既是领导工作的基本方法，又是制定政策的价值取向。“稳”和“进”辩证统一，“稳”是基础和前提，“进”是目标和方向，必须把“稳”和“进”作为一个整体来把握，既要立足于“稳”，保持经济平稳增长；又要着眼于“进”，在高质量发展上取得新进展，以稳求进、以进促稳，推动全省经济社会不断向前发展。

　　同时，注意抓住“四个关键”：一是始终把发展扭住。发展始终是第一要务，是解决一切问题的总钥匙。任何时候都要坚持发展硬道理，牢固树立新发展理念，坚持高质量发展根本方向，增强定力信心，突出科学发展，补齐发展短板，优化发展环境，保持经济社会持续健康发展良好态势。二是着力把重点抓住。始终坚持以深化供给侧结构性改革为主线，推动经济由高速增长转向高质量发展，全面贯彻落实“八字方针”，深刻把握巩固成果是前提、增强活力是基础、提升水平是重点、畅通循环是目标，强化改革推动、开放带动、创新驱动，增强高质量发展动力活力。三是坚决把风险管住。牢固树立底线思维，紧盯经济发展、政治安全、社会稳定、自然灾害、公共事件、安全生产六大领域风险，着力解决可能影响高质量发展的突出问题，坚决守住不发生系统性区域性风险的底线，夯实高质量发展基础。四是切实把民生兜住。坚持以人民为中心的发展思想，持续加大民生投入，增强社会保障能力，促进就业稳定扩大，不断提高居民收入，切实办好民生实事，让改革发展成果更多更公平惠及人民群众。

　　今年重点抓好十个方面的工作：

　　（一）着力扩需求稳增长，保持经济健康运行。坚持精准出招应变，努力扩大市场需求，支持实体经济发展，保持经济稳中向好态势。

　　激活区域市场需求。抢抓国家促进形成强大国内市场的机遇，加快消费升级，挖掘健康、育幼、体育、休闲、文化、信息等领域和传统服务业消费潜力，落实个人所得税专项附加扣除政策，培育家政服务、现代生活用品等新的消费热点。实施健康养老示范工程，加快城市社区养老服务设施建设；完善旅游设施，提升服务品质，推进旅游业转型发展；加快发展农村电商，开拓农村消费市场；开展服务业质量提升行动，严厉打击侵害消费者权益行为，改善消费环境。扩大有效投资，聚焦产业转型升级、城市功能配套、生态环境建设、交通基础设施、农村水利工程等重点领域和薄弱环节，开工建设一批重大项目，确保全社会固定资产投资稳定增长。稳定对外贸易，引导企业多元化开拓国际市场，积极培育引进外贸综合服务企业，加强外贸优势产业基地建设，保持货物进出口稳定增长。

　　稳定工业经济增长。切实加强运行调度和监测预警，强化要素保障，搞好企业服务，确保工业平稳运行。尤其要把推动制造业高质量发展作为促进工业稳定增长的重要抓手，开展智能制造引领、重点技术攻坚、传统产业提质、新兴产业培育四大行动，实施上汽乘用车二期、同人铝业高端铝合金板等一批重大项目，支持宇通客车、森源电气等一批骨干企业，培育平顶山尼龙新材料、许昌远东传动轴智能制造等一批产业园区，重点支持主营业务收入100亿元以上大型制造业企业和50亿元以上高成长制造业企业发展。同时，加快推进现代服务业和先进制造业深度融合，为巩固稳中有进发展态势夯实基础。

　　促进非公经济发展。认真贯彻习近平总书记在民营企业座谈会上的重要讲话精神，做到“两个毫不动摇”。培育壮大民营企业家队伍，大力弘扬企业家精神。构建亲清新型政商关系，主动为民营企业排忧解难，畅通企业家投诉渠道，依法保护其合法权益。营造公平环境，消除民营企业在准入许可、工程投标、银行融资等方面的不公平待遇。落实支持政策，打破各种“弹簧门”“玻璃门”“旋转门”，让企业家既能看得见、又要摸得着。引导提升企业素质，推进民营企业制度创新、科技创新、产品创新、模式创新，促进二次创业和转型发展。

　　（二）贯彻落实“八字方针”，促进经济提质增效。按照“八字方针”总要求，深化供给侧结构性改革，加快发展方式转变、经济结构转型、增长动能转换。

　　深化供给结构改革，巩固“去降补”成果。坚持去化过剩产能劲头不松，严格执行环保、能耗、质量、安全、技术等标准，加快拆除已实施置换的低效钢铁设备，积极化解水泥、电解铝等过剩产能，淘汰改造落后煤电机组150万千瓦左右。坚持处置“僵尸企业”力度不减，做好债务化解、破产审理、税收支持、资产处置等工作。坚持扶持结构调整政策不变，加大对“破、立、降”奖补支持力度，落实好去产能职工安置、矿权土地、债务处置等政策，形成正向激励和优胜劣汰的政策导向。

　　改善优化营商环境，增强微观主体活力。打好优化发展环境“组合拳”，营造便利高效政务环境、开放公平市场环境、公正透明法治环境、诚实守信信用环境。加强部门协同，打通政策落实“最后一公里”。启动营商环境评价，把评价权交给服务对象，让服务对象评价政府部门。加快诚信体系建设，完善守信褒奖、失信联合惩戒机制，坚决纠正一些地方和部门“新官不理旧账”的违约做法。落实中央更大规模减税降费政策，实行普惠性减税和结构性减税相结合，对小微企业和科技型初创企业实施普惠性税收减免，清理规范地方收费项目，全面实施收费项目清单制，继续实行省定涉企行政事业性零收费，加大力度降低企业社保费率，让企业有实打实的获得感。

　　继续实施“三大改造”，提升产业链水平。以产业高端化、技术尖端化、质量顶端化为目标，加快构建现代产业体系。持续实施12个省定重点产业转型发展专项方案，纵深推进绿色、智能、技术改造，深入开展五大传统产业转型、十大战略新兴产业发展行动及智能制造和工业互联网发展三年行动。完善数字基础设施，推动资源整合和开放共享，加快大数据产业发展。深入开展“质量标杆进企业”活动，弘扬劳模精神和工匠精神，支持企业增品种、提品质、创品牌。推进产业集聚区二次创业，更加注重产业建链、补链、延链，打造主导产业优势明显的产业集群。

　　积极完善市场体系，畅通经济循环系统。着力破解生产主体和市场错配、生产和消费脱节、金融和实体失衡的难题，畅通生产、流通、分配、消费循环。从正面清单转向负面清单、从分散市场转向统一市场、从差别待遇转向同等待遇，加快建设统一开放、竞争有序的现代市场体系。开展重点领域综合金融服务行动，加快建设民营企业金融服务系统，加大企业上市培育力度，推广建设银行“云税贷”等新型金融产品，加快发展普惠金融、科技金融、绿色金融，提高金融服务实体经济能力。强化产销、产需、产运对接，大力发展冷链物流、快递物流、电商物流，创新多式联运，加快构建覆盖城乡的分拨配送物流网络。

　　（三）持续推进三大攻坚，着力夯实发展基础。把打好三大攻坚战摆在全局更加突出的位置，打好重点战役，全力攻坚，务求实效。

　　防范化解风险。把国有企业去杠杆作为重中之重，推进市场化债转股签约项目落地，探索开展民营企业债转股，引导企业加大股权融资力度，降低企业经营风险。全面加强地方金融监管，发挥好企业债权人委员会作用，有效防控企业债券违约、股票质押平仓、担保圈、网络借贷、非法集资等风险隐患，高度关注房地产领域金融风险。依法规范政府举债行为，实施“八个一批”解决方案，稳妥有序化解地方债务风险。压实安全生产责任，全面推行双重预防体系建设，健全应急管理体系，强化源头管控治理，坚决防范遏制各类事故发生。增强防洪抗灾能力，防范自然灾害风险。做好突发公共事件预防处置，防范社会风险。

　　推进精准脱贫。坚持精准方略，把脱贫质量放在首位，加大深度贫困县村、特殊贫困群体、重点贫困县区脱贫攻坚力度，重点解决好“两不愁、三保障”问题。统筹贫困县村和一般县村、地区帮扶和家庭帮扶、经济发展和社会发展、内生动力和外部动力，继续打好“四场硬仗”，开展“六大行动”，实施“四项工程”，打好脱贫攻坚总攻战。建立脱贫正向激励和稳定脱贫长效机制，减少和防止贫困人口返贫。严格落实市县主体责任、部门协调责任、乡村包干责任、驻村帮扶责任，确保14个国定贫困县脱贫摘帽。完成20万黄河滩区居民搬迁。

　　抓好污染防治。扭住改善环境质量这个核心，扎实开展中央环境保护督察“回头看”反馈问题整改，聚焦打赢蓝天、碧水、净土保卫战，深入实施“四大行动”。优化调整“四个结构”，持续抓好“六控”，推进郑州市主城区煤电机组清零和洛阳市主城区煤电机组基本清零，确保PM2.5平均浓度和优良天数比例完成国家考核目标。严格落实河长制湖长制，加强饮用水水源地保护，逐步消除城区黑臭水体。加强重金属污染、农业面源污染防治和固体废物处理，推进受污染耕地安全利用和治理修复，严格保护未污染土壤。严格落实党政领导责任、企业主体责任、部门监管责任，确保环境质量持续改善。

　　（四）加大力度深化改革，激发经济社会活力。加快市场化改革，让市场主体不断壮大起来，让生产要素自由流动起来，让改革效力充分释放出来。

　　深化国有企业改革。全力打好国企改革“扫荡战”，在做好“僵尸企业”处置后续工作的同时，持续深化产权结构、组织结构、治理结构改革，深入推进混合所有制改革，建立规范的现代企业制度。持续完善国有资产监管，理顺国有金融资本管理体制机制，改组组建国有资本投资、运营公司，加快实现由管企业向管资本转变。

　　深化财税体制改革。推进省以下分领域财政事权和支出责任划分改革，制定按市县财力状况分档分担资金的改革方案，进一步理顺省与市县财政分配关系。加快构建现代预算制度，全面实施预算绩效管理，提高财政资金配置效率和使用效益。

　　深化投融资体制改革。继续优化投资项目审批，大力推广多评合一、企业投资项目承诺制等模式，一般性企业投资项目全流程审批时限压减至100个工作日以内。用好用足地方政府债券政策，支持发行企业债券、双创专项金融债券和孵化专项债券。发挥好地方政府融资担保体系作用，规范发展政府和社会资本合作项目。用好政府投资杠杆撬动作用，充分激发民间投资活力。

　　深化农村综合改革。继续推进农村承包地“三权分置”改革，探索农村宅基地改革。加快农村集体产权制度改革，完成农村集体资产清产核资，发展新型集体经济。持续深化、有序推进农村土地征收制度改革和农村集体经营性建设用地入市改革，加快建立城乡统一的建设用地市场。深化农村金融改革，强化农业保险保障作用。持续推进水利、供销社、农场、林权等改革。

　　健全生态环保体制。完善月度生态补偿、绿色环保调度、污染物排放许可证等制度。实施自然资源资产产权制度和有偿使用制度改革。推进省以下生态环境机构监测监察执法垂直管理体制改革，抓好生态环境保护综合行政执法改革。

　　推进政府机构改革。继续深入推进省级机构改革，改革机构设置，优化职能配置，提高行政效能，加快完成市县机构改革。深入推进政府“放管服”改革，把“一网通办”前提下的“最多跑一次”改革落到实处，加强“双随机、一公开”监管。加快建设全省一体化政务服务平台和“互联网+监管”系统，打破信息孤岛，加快信息共享，提升政府管理数字化水平，最大限度利企便民。

　　同时，抓好教育、医疗、警务管理、城市综合执法等改革。

　　（五）坚持扩大对外开放，提升经济发展优势。坚持以郑州航空港经济综合实验区为龙头，统筹“五区”联动、“四路”协同，深度融入“一带一路”建设，推动全方位高水平开放。

　　放宽市场准入，拓展开放领域。贯彻国家大幅度放宽市场准入部署，全面落实准入前国民待遇加负面清单管理制度，强化科技、金融、产业、贸易合作，在农业、教育、医疗、文化、电信、制造业等领域谋划一批重大项目，吸引外商投资。高质量办好第十三届中国（河南）投洽会等活动，精心组织参加第二届中国进口博览会。

　　打造平台载体，提升开放水平。高水平建设中国（河南）自由贸易试验区，持续深化五大服务体系建设，加快建立与国际贸易投资规则相衔接的制度体系。发挥各类口岸作用，建设海外仓及物流分拨中心。全力拓展“四条丝路”，做大做强郑州—卢森堡航空“双枢纽”，提升中欧班列运营水平，支持跨境电商发展，推动EWTO核心功能集聚区建设，拓展海铁联运班列线路。

　　健全体制机制，优化开放环境。稳步推进外资管理体制改革，加快复制推广自贸区经验做法，完善外资企业设立商务备案与工商登记“一口办理”，建立外商投资企业投诉工作机制，严格企业知识产权保护，加快形成法治化、国际化、便利化营商环境。加快国际贸易“单一窗口”建设，提升通关效率，降低进出口环节收费，优化出口退税服务，提升贸易便利化水平。

　　（六）深入推进科技创新，加快增长动能转换。把创新作为高质量发展的第一动力，以郑洛新自创区建设为龙头，加快提升创新发展水平。

　　完善科技创新体系。聚焦培育“四个一批”，实施创新龙头企业提升引领、高新技术企业倍增、“科技小巨人”企业培育工程。坚持引进外部人才和培育本地人才并重，落实人才政策待遇，重视解决住房、子女教育等实际问题，建设高层次创新人才团队。争创国家重点实验室、掘进机械国家技术创新中心等高端创新平台，加快建设国家生物育种产业创新中心、国家超算中心，发展新型研发机构。实施“十百千”转型升级创新专项，抓好生物育种、通信技术、超级电容、工业CT等项目，加快实施盾构、燃料电池客车、高铁轴承、光互连芯片、铜合金新材料创新引领专项。

　　提升科技创新能力。落实与科技部会商成果，举办“中国·河南开放创新暨跨国技术转移大会”，培育壮大技术转移转化市场，探索开展订单式研发和成果转化，实行重大创新需求面向国内外揭榜攻关，组织企业揭榜转化国内外重大科技成果。持续促进“四个融合”，用军民融合之力解决关键核心技术“卡脖子”问题，引导企业、高校、科研院所围绕产业链布局创新链，提升产业技术创新战略联盟建设水平。在国家级高新区复制推广自创区管理体制改革经验，营造创新生态。

　　优化科技创新服务。加大财政投入，引导企业增加研发投入，支持企业、高校、科研院所构建开放、协同、高效的共性技术平台，完善大型科研仪器设备共享机制。建设省科技信息综合服务平台，支持发展一批科技服务机构。深化项目评审、人才评价、机构评估改革，落实国家科技成果转化激励改革举措，建立试错、容错、纠错机制，让创新者敢于创新、安于创新、乐于创新。

　　（七）统筹推进城乡建设，促进区域协调发展。坚持把城乡作为一个整体，优化国土空间布局，统筹实施新型城镇化和乡村振兴战略，加快形成城乡融合、区域协调发展新格局。

　　以“五个振兴”为路径，大力实施乡村振兴战略。坚持乡村振兴战略总要求，围绕农业强、农村美、农民富，加快推进产业、人才、文化、生态、组织“五个振兴”。坚持质量兴农、绿色兴农、品牌强农，稳定粮食产能，推进“四优四化”，加快绿色食品业转型升级，促进农村一二三产融合发展，提高农业效益，增加农民收入。加快驻马店国际农产品加工产业园建设。创新农业经营主体，培育新型职业农民。学习浙江经验，坚持规划先行，改善农村人居环境，抓好垃圾污水处理、“厕所革命”、废弃物资源化利用、村容村貌提升。塑造乡风文明，开展移风易俗，保护好农村传统文化、乡土文化。加强农村基层组织建设，深化拓展“四议两公开”工作法，创新乡村治理机制，提高乡村治理效能。开展全域国土综合整治，深入推进“大棚房”与农地非农化问题治理。毫不松懈抓好非洲猪瘟防控工作。

　　以推进城市高质量发展为目标，全面开展百城建设提质。推动百城建设提质工程由试点先行到全面铺开，着力推进城市规划、建设、管理、经营高质量，促进城市由注重外延扩张转向注重内涵提质。谋划实施一批道路交通、污水垃圾处理、热力燃气、地下综合管廊、地下空间综合体、地下停车场等市政补短板项目，加快建设一批教育、医疗、养老、文化、体育等公共服务项目，全面开展省辖市城区生活垃圾分类工作，规划建设一批生活垃圾焚烧处理和餐厨废弃物处理设施，有序推动城市老旧小区综合功能改造。积极打造智慧城市。加强绿色园林城市建设，创建一批国家级、省级园林城市和园林城镇。

　　以中心城市建设为引领，加快中原城市群一体化。优化城市空间布局，加快形成以郑州为中心、洛阳为副中心、多极支撑发展的大格局。支持郑州建设国家中心城市，加快郑州大都市区建设，带动开封、许昌、新乡、焦作等周边城市融合发展。推动洛阳中原城市群副中心城市建设，增强其对陕东、晋南地区的资源聚合和区域辐射作用，打造带动全省经济发展新的增长极。统筹推进区域性中心城市特别是毗邻兄弟省份的城市竞相发展，增强南阳对鄂西北、陕东南，信阳对鄂北、皖西，商丘对鲁西南、皖北，安阳对冀南、晋东的影响，发挥其对周边要素的吸纳集聚作用。其他省辖市要立足在全省大局中的定位，做到错位发展、特色发展。加快推进县域经济高质量发展，规范引导特色小镇和小城镇布局建设。统筹户籍制度改革和城镇基本公共服务均等化，实现农业转移人口在城镇落户200万人以上。

　　（八）加快实施重大工程，强化基础支撑能力。基础设施既是高质量发展的支撑基础，也是可持续发展的重要条件。必须抢抓国家基础设施领域补短板的政策机遇，加快实施一批重大项目，支撑区域经济发展。

　　加快构建便捷畅通的综合交通体系。以高速公路、骨干铁路和航空枢纽为主体，建设贯通南北、连接东西的现代立体综合交通体系。实施投资1000亿元以上、总长1000公里以上的高速公路“双千工程”，开工建设栾川至卢氏高速公路等一批项目，建成周口至南阳等3条高速公路。加快郑州高铁南站枢纽和三洋铁路河南段建设，建成郑万、郑阜、商合杭高铁和蒙华铁路河南段，开工郑济高铁濮阳至济南段，推进呼南高铁前期工作。开工郑州机场三期北货运区、中国邮政航空邮件处理中心等项目。推进沙颍河、淮河等内河水运项目建设，构建通江达海水运网络。完善综合枢纽场站、集疏运通道和区域物流节点等重点物流基础设施，加快建设中国（郑州）有色金属国际物流港产业园、中储洛阳综合物流产业园仓储配送中心等一批重大项目，推进一批多式联运示范工程，促进铁、公、空、水等多种运输方式无缝衔接。

　　加快构建兴利除害的现代水网体系。深入推进“四水同治”，加快十大水利工程建设，构建旱引涝排、上灌下补、内连外通、丰蓄枯补、调洪防灾的现代水网体系。实施宿鸭湖水库清淤扩容工程、卫河共渠和洪汝河治理工程，提高防洪除涝能力。扩建黄河下游贯孟堤工程，从根本上改善封丘倒灌区防洪条件和发展环境。联动实施引江济淮和赵口灌区二期工程，充分利用现有水系条件，形成长江、淮河、黄河互联互通互补的水网格局，有效解决豫东南水资源短缺问题。建设大别山革命老区引淮供水灌溉工程，助推革命老区脱贫攻坚。开工建设小浪底南岸、北岸灌区和西霞院水利枢纽输水及灌区工程，解决两岸人民守着黄河没水用的问题，让人民群众喝上安全水、用上自来水、补上生态水。

　　加快构建低碳高效的能源支撑体系。持续推进能源外引通道建设，实现省内互联互通，优化能源结构布局。全面推动青电入豫工程建设，提高吸纳清洁外电能力；加快驻马店东等500千伏输变电工程建设，缓解豫南地区供电能力薄弱问题。在全面完成新一轮农网改造升级基础上，继续加大城乡配电网投资，提升农村电网保障能力。加快发展清洁电源，新增可再生能源发电装机180万千瓦。建成鄂安沧输气管道濮阳支线等能源项目，力争开工西气东输三线中段，全面畅通省内输气网络。力争完成洛炼扩能改造项目一期和日照—濮阳—洛阳原油管道主体工程，持续推进洛炼改扩建项目，力争开工洛阳—新郑机场航空煤油管道，提升省内高端油品供应能力。

　　加快构建绿色循环的生态环境体系。深入开展山水林田湖草一体化生态保护和修复，统筹建设森林、湿地、流域、农田、城市五大生态系统。加强南水北调中线水源地保护，实施南太行地区生态保护修复工程，启动沿黄生态带、郑州黄河中央湿地公园、中华生物园和伏牛山世界植物大观园等重大项目。加快国土绿化提速行动，建设森林河南，完成造林249万亩、森林抚育400万亩，推进山区森林化、平原林网化、城市园林化、乡村林果化、廊道林荫化和庭院花园化，提升生态环境承载力。

　　加快构建高效泛在的信息网络体系。聚焦网络强省建设，实施一批基础性、全局性、战略性的重大信息网络基础设施项目。推进下一代互联网规模部署和千兆城市建设，加快5G商用步伐。持续实施郑州直联点提升工程，加快中国联通中原数据基地二期、中国移动（河南）数据中心一期等重点项目建设，巩固提升河南信息集散中心和网络交换枢纽地位。

　　（九）大力发展社会事业，提高公共服务水平。坚持抓重点、强弱项、提质量，不断优化公共服务供给。

　　坚持教育优先发展。落实立德树人根本任务，促进学生德智体美劳全面发展。继续实施第三期学前教育行动计划，促进学前教育普及普惠发展。持续建设农村寄宿制和小规模学校。规范校外培训机构。改善普通高中办学条件。深入推进产教融合、校企合作和实训基地建设，发展一批高水平职业院校。推动高等教育内涵发展，加快郑州大学、河南大学“双一流”建设，提升特色骨干大学办学水平，推动符合条件的普通高校向应用技术型转变。积极推进国内外名校来豫合作办学。做好民办教育分类管理。加强教师队伍建设，改善教师工作生活条件，提高集中连片特困地区乡、村、教学点教师生活补助标准，使教师成为最受社会尊重的职业。加强综合治理，维护校园安全。健全学生资助制度，使更多孩子享受到公平而有质量的教育。

　　提高人民健康水平。完善公共卫生服务体系，实施健康素养促进行动，努力提高人均预期寿命。持续推动六大国家区域医疗中心建设，扩充优质高端医疗资源。全面推进县域综合医改。加快发展“互联网+医疗健康”，促进优质资源互联共享。做实家庭医生签约服务，完成乡村医生全员培训。推行按病种付费为主的复合付费方式。加快推进现代医院管理制度建设。大力支持社会办医。实施中医药传承创新工程。加强生育全过程健康服务，提高妇幼保健水平。广泛开展全民健身活动，推进中原网球中心二期等公共体育设施建设。深化食品安全省建设，加强疫苗等药品安全监管，让群众吃得更放心、用药更安全。

　　促进文化发展繁荣。深入推进华夏历史文明传承创新区建设，加快打造文化强省。积极践行社会主义核心价值观，大力弘扬焦裕禄精神、红旗渠精神、愚公移山精神。抓好新时代文明实践中心建设，深化群众性精神文明创建。繁荣艺术创作，丰富文化生活。推进大运河（河南段）文化带建设，加快省科技馆、中原考古博物院建设。加强文物保护利用，建成二里头遗址博物馆，开工建设殷墟国家考古遗址公园，实施隋唐洛阳城、宋都古城等一批保护利用项目。支持太极拳项目申遗。加快文化产业发展，培育新型文化业态。促进文化旅游融合发展，叫响“中华源·老家河南”品牌。繁荣发展哲学社会科学，建设新型智库。深入开展“扫黄打非”。打造新型主流媒体，推进媒体融合发展。提高网络治理能力，打造清朗网络空间。

　　（十）加大民生保障力度，切实增进人民福祉。始终坚持以人民为中心的发展思想，尽力而为、量力而行，完善制度、守住底线，精心做好各项民生工作。

　　办好民生实事。积极优化财政支出结构，继续压减政府一般性支出，腾出更多财力用于办好民生实事。一是新增城镇就业110万人。二是参加城乡居民基本医保的80岁以上高龄老人住院报销比例在现行政策基础上提高5个百分点，实施80岁以上老人高龄津贴制度。三是继续免费开展妇女宫颈癌和乳腺癌筛查、预防出生缺陷产前筛查和新生儿疾病筛查。四是实施残疾儿童康复救助。五是开展农村人居环境“千村示范、万村整治”工程，完成300万户农村户用卫生厕所改造，完成剩余45个县（市、区）农村垃圾治理省级达标验收任务。六是持续改善大气环境质量，全省空气优良天数比例达到59.5%。七是实施“百县通村入组工程”，新改建农村公路5000公里以上；开展“万村通客车提质工程”，所有具备条件的行政村通客车率达到100%。八是新建和改扩建城乡幼儿园1000所，新增学位10万个。九是完成3000个电网薄弱行政村电网改造。十是加快县（市）人民医院提质升级。

　　稳定扩大就业。把稳就业放在更加突出位置，加强省内就业统筹和岗位余缺调剂，实施失业保险援企稳岗“护航行动”。积极推进公共就业服务专项活动，持续实施全民技能振兴工程，抓好高校毕业生、退役军人等重点群体就业，稳定农村劳动力转移就业，做好“僵尸企业”处置和过剩产能化解中职工安置工作，开展各类职业培训300万人次，新增农村劳动力转移就业40万人。支持农民工等群体返乡下乡创业，新增创业人员20万左右。

　　加强社会保障。强化兜底保障功能，大力实施全民参保计划，实现城乡居民基本养老保险基金省级管理、企业养老保险基金省级统收统支，继续提高城乡居民基础养老金最低标准，继续调增企业和机关事业单位退休人员基本养老金。实施工伤保险基金省级统筹。完善城乡居民医保门诊统筹制度，提高基本医保、大病保险、困难群众大病补充医疗保险保障水平。提高城乡低保、特困供养、孤儿养育等保障标准。加强残疾人权益保障。坚持房子是用来住的、不是用来炒的定位，夯实城市政府主体责任，推动房地产市场平稳健康发展，完善住房市场体系和住房保障体系，新开工棚改安置房15万套左右，更好解决群众住房问题。

　　创新社会治理。学习推广“枫桥经验”，全面加强基层社会治理。开展阳光信访、责任信访、法治信访，发挥信访工作联席会议作用，促进人民群众合理合法利益诉求及时、就地解决。深入开展普法教育，推进法制宣传教育进校园、进农村、进社区、进机关、进企业、进家庭。加强社会治安防控体系建设，高质量推进“一村（格）一警”工作，依法打击各种违法犯罪活动，推动扫黑除恶专项斗争纵深发展，严厉打击敌对势力、邪教组织、非法宗教活动向农村地区的渗透。扎实做好庆祝新中国成立70周年安保等工作，维护社会大局和谐稳定。

　　加强国防教育、国防动员和国防后备力量建设，支持国防和军队改革，支持驻豫解放军、武警部队、预备役部队建设，抓好民兵和人民防空建设，深入开展双拥共建，加强退役军人服务保障体系建设，深度推进军民融合发展，巩固军政军民团结。全面贯彻党的民族政策和宗教工作基本方针，加强民族宗教工作基层基础建设，办好第十一届全国少数民族传统体育运动会，促进民族团结、宗教和睦。支持工会、共青团、妇联等人民团体更好发挥桥梁纽带作用。做好外事、侨务、港澳、对台工作。加强统计工作，搞好第四次经济普查。抓好参事、史志、文史、档案、气象、地震、测绘、地质、邮政、援疆等工作。

　　三、加强政府自身建设，全面提高履职能力和服务水平

　　新形势对政府工作赋予了新的使命、提出了新的要求。各级政府要不辱时代使命，不负人民重托，更加注重加强自身建设，提升治理能力，加快建设人民满意的法治政府、创新政府、廉洁政府和服务型政府。

　　（一）树牢“四个意识”，坚定“两个维护”。“两个维护”是党的十八大以来我们党的重大政治成果和宝贵政治经验，是我们党最重要的政治纪律和政治规矩。无论形势多么复杂，挑战多么严峻，任务多么艰巨，只要我们坚决做到“两个维护”，就一定能够不断从胜利走向胜利。政府工作人员要牢固树立“四个意识”，坚定“四个自信”，任何时候都要坚决维护习近平总书记党中央的核心、全党的核心地位，坚决维护党中央权威和集中统一领导，真正把“两个维护”内化于心、外化于行，主动对标、自觉看齐，在政治立场、政治方向、政治原则、政治道路上同以习近平同志为核心的党中央保持高度一致，听从党中央号令，紧跟党中央步伐，有令则行、有禁则止，不讲条件、不搞变通，确保习近平总书记重要指示批示和党中央决策部署贯彻落实不走样，决不允许不敬畏、不在乎、喊口号、装样子。

　　（二）弘扬法治精神，严格依法行政。各级政府必须坚持依法施政、依法行政、依法办事，严格按照法定权限和法定程序行使权力，自觉做到法无授权不可为、法定职责必须为，善于运用法治思维和法治方式深化改革、推动发展、化解矛盾、维护稳定。坚持依法决策，健全完善程序，充分发扬民主，正确实行集中，防止个人专断，对重大民生政策出台、重大公共项目建设要依法公示听证，进行深入咨询，组织专家论证，进一步提高政府决策科学化、民主化、法治化水平。推进政务公开，加强政策宣传解读，主动回应社会关切。自觉接受人大法律监督、工作监督和政协民主监督，自觉接受监察机关的监督，主动接受社会和舆论监督。规范行政执法，心中高悬法律的明镜，手中紧握法律的戒尺，忠诚履行宪法和法律赋予的职责，防止任性用权，纠正粗暴执法，决不允许以权代法、以权压法、逐利违法、徇私枉法。

　　（三）牢记为民宗旨，保持清正廉洁。权力来自人民必须服务人民。政府工作人员要树立以人民为中心思想，全心全意为人民服务、真心实意为人民办事，走好新形势下的群众路线，始终与人民群众站在一起、想在一起、干在一起，既“身入”基层，又“心到”群众，想群众之所想、急群众之所急、忧群众之所忧。要严格遵守廉洁自律准则，筑牢思想防线，保持清醒头脑，始终敬畏人民、敬畏组织、敬畏法纪，在各种诱惑和“围猎”面前，自觉划清边线、不碰红线、守住底线，做到忠诚、干净、担当。坚决贯彻中央八项规定及实施细则精神，驰而不息正风肃纪，坚定不移反腐倡廉，深入抓好以案促改，坚决铲除滋生腐败的土壤。

　　（四）提高能力素质，锤炼过硬本领。肩负新使命，战胜新挑战，既要有谋事的大智慧，还要有成事的真功夫。政府工作人员要提高理论素养，坚持用习近平新时代中国特色社会主义思想武装头脑，不断汲取这一科学理论蕴含的强大真理力量、信仰力量和实践力量，指导实践、推动工作。提高政治能力，增强政治敏锐性、政治鉴别力，做到眼睛亮、见事早、行动快，对容易诱发政治问题的敏感因素，准确判断、快速处置。提高专业能力，解放思想、开阔视野，防止思想僵化、知识老化、本领弱化，注重实践锻炼，加快知识更新，涵养专业素质。提高斗争能力，增强斗争意识，发扬斗争精神，讲究斗争艺术，在推进重点工作中积累经验，在解决重大矛盾中经受考验，在完成重要任务中增长才干，使政府工作体现时代性、把握规律性、富于创造性，不断增强政府治理能力和服务水平。

　　（五）始终恪尽职守，勇于担当作为。新的征途不会平坦，前进道路充满挑战。做好今年的工作，既考验我们的判断能力，更检验我们的担当精神。我们要以对发展负责、对历史负责、对人民负责的态度，始终保持锐意进取、永不懈怠的精神状态和敢闯敢干、一往无前的奋斗姿态，不回避矛盾，不惧怕挑战，不推卸责任，勇于挑最重的担子、啃最硬的骨头、接最烫的山芋，敢于承担风险，敢于迎难而上，勤勉重干，真抓实干。反对空泛表态、应景造势、敷衍塞责、沽名钓誉，坚决纠正脱离实际的官僚主义、空喊口号的形式主义、简单粗暴的命令主义。各级政府要坚持严管与厚爱结合、激励和约束并重，理解基层工作的难处，关心基层干部的生活，为担当的干部担当，对负责的干部负责，让干事的干部有想头，使成事的干部有奔头，营造一种人人尽责、个个实干，奋发向上、担当作为的良好局面。

　　各位代表！

　　奋斗创造幸福，实干成就梦想。让我们在省委领导下，更加紧密地团结在以习近平同志为核心的党中央周围，高举习近平新时代中国特色社会主义思想伟大旗帜，同心同德，砥砺奋进，谱写新时代中原更加出彩新篇章，以优异成绩迎接中华人民共和国成立70周年！
